# Marginal diffusion slope as a prognostic imaging biomarker of infiltrating phenotype in glioblastoma; A cancer imaging biomarker roadmap study

**DOI:** 10.1093/noajnl/vdag028

**Published:** 2026-02-17

**Authors:** Antoine Vallatos, Haitham F I Al-Mubarak, Joanna L Birch, Yusuf Icer, Catherine McBain, David J Coope, Konstantina Karabatsou, Samantha J Mills, Geoff J M Parker, William M Holmes, Anthony J Chalmers, Alan Jackson, Adam Waldman, Gerard Thompson

**Affiliations:** Edinburgh Neuro-Oncology Translational Imaging Research (ENTIRe), Institute for Neuroscience and Cardiovascular Research, University of Edinburgh, Edinburgh, UK; Glasgow Experimental MRI Centre, School of Psychology and Neuroscience, University of Glasgow, Glasgow, UK; School of Cancer Sciences, University of Glasgow, Glasgow, UK; Glasgow Experimental MRI Centre, School of Psychology and Neuroscience, University of Glasgow, Glasgow, UK; University of Misan, Amarah, Iraq; Cancer Research UK Scotland Institute, Scotland, UK; School of Cancer Sciences, University of Glasgow, Glasgow, UK; Edinburgh Neuro-Oncology Translational Imaging Research (ENTIRe), Institute for Neuroscience and Cardiovascular Research, University of Edinburgh, Edinburgh, UK; University of Manchester, Manchester, UK; The Christie Hospital, Manchester, UK; University of Manchester, Manchester, UK; Northern Care Alliance, Manchester, UK; University of Manchester, Manchester, UK; Northern Care Alliance, Manchester, UK; Walton Centre for Neurology and Neurosurgery, Liverpool, UK; Centre for Medical Image Computing, Department of Medical Physics and Biomedical Engineering, University College London, London, UK; Bioxydyn Limited, Manchester, UK; Glasgow Experimental MRI Centre, School of Psychology and Neuroscience, University of Glasgow, Glasgow, UK; Cancer Research UK Scotland Institute, Scotland, UK; School of Cancer Sciences, University of Glasgow, Glasgow, UK; University of Manchester, Manchester, UK; Edinburgh Neuro-Oncology Translational Imaging Research (ENTIRe), Institute for Neuroscience and Cardiovascular Research, University of Edinburgh, Edinburgh, UK; Cancer Research UK Scotland Institute, Scotland, UK; Edinburgh Neuro-Oncology Translational Imaging Research (ENTIRe), Institute for Neuroscience and Cardiovascular Research, University of Edinburgh, Edinburgh, UK; Cancer Research UK Scotland Institute, Scotland, UK

**Keywords:** diffusion MRI, glioblastoma, imaging biomarker, ­infiltration, prognosis

## Abstract

**Background:**

Conventional MRI protocols fail to probe marginal tumour infiltration in glioblastoma, hindering surgery and radiotherapy planning. This study aimed to demonstrate development and biological validation of a putative imaging biomarker (IB) for characterising glioblastoma infiltration, following principles outlined in the cancer imaging biomarker roadmap.

**Methods:**

This IB is based upon spatial change in apparent diffusion coefficient (ADC) measures across a macroscopic tumour boundary. We systematically assessed whether ADC slope at the tumour margin (marginal diffusion slope-MDS) could (a) describe an underlying infiltrative phenotype based on reported links between ADC and tumour cellularity validated using a preclinical model, and (b) predict clinical outcome in a single-centre exemplar prospective human cohort study.

**Results:**

Preclinical results showed a strong, spatially-resolved, negative correlation between marginal ADC and underlying tumour cell density in coregistered MRI-histology datasets from a glioblastoma model. Clinical results (*n* = 18) showed a positive linear correlation between MDS and clinical outcome, with higher MDS (i.e., steeper marginal ADC slope) associated with longer survival (Pearson’s correlation was 0.636, *P* < .005). Cox proportional hazard analysis yielded a survival model (*P* = .013) with MDS significantly associated with overall survival (OS) controlling for age (age *P* = .35, MDS *P* = .010). The hazard ratio for each MDS standard deviation was 0.47 (range 0.25–0.89), indicating that higher MDS predicts longer survival.

**Conclusions:**

In alignment with the Imaging Biomarker Roadmap consensus, we biologically validated MDS as a biomarker of infiltrative phenotype in a preclinical model and demonstrated its predictive value for OS in humans as a prelude to larger clinical validation studies.

Key Points

- GBM infiltration biomarker validated: modelling, preclinical, clinical.
- MDS offers a quantitative measure of tumour boundary infiltration.
- MDS shows promise as a prognostic biomarker for glioblastoma patient survival.

Importance of the Study

Glioblastoma (GBM) is highly infiltrative, with microscopic disease extending beyond visible tumour margins, leading to near-universal recurrence and poor prognosis. Current clinical imaging predominantly relies on T1-weighted (T1w) intravenous gadolinium-based contrast agent (GBCA) enhancement, which poorly reflects this infiltrative burden. This study addresses the initial steps towards the urgent unmet need for a robust, clinically translatable imaging biomarker of GBM infiltration. We systematically evaluate Marginal Diffusion Slope (MDS), derived from readily available diffusion-weighted imaging, demonstrating its link to cellular infiltration in preclinical models and its predictive value for overall patient survival. MDS offers a non-invasive, quantitative tool to better characterize GBM infiltration, potentially informing treatment planning, improving patient stratification, and facilitating future therapeutic strategies.

Glioblastoma (GBM) is the most aggressive and prevalent primary brain tumour. It is the commonest cause of cancer-related death in under-40s, with a median survival under 2 years and single-digit 5-year survival, leading to one of the highest rates of years of life lost across all cancers.

The mainstay treatment for GBM is surgery followed by temozolomide chemoradiotherapy.^1^ Provisional diagnosis, surgical and radiotherapeutic treatment, and assessment of response are all dependent on imaging, but despite decades of research, clinical imaging for diagnosis, therapy planning, and response assessment in GBM is predominantly based on structural magnetic resonance imaging (MRI), which has changed relatively little in 30 years. Although macroscopic disease is often identified by contrast enhancement on T1-weighted images, this epiphenomenon of vascular leakiness does not directly reflect the presence of tumour; contrast enhancement can be seen without a tumour (e.g., radiation necrosis) and infiltrating tumour may not produce detectible enhancement.

Overall survival (OS) remains dismal even with imaging and fluorescence-guided maximal safe resection practices; this is largely attributed to the inevitable presence of micro-invasive disease beyond the enhancing tumour.^2-4^ While this has been confirmed by biopsy and post-mortem studies, it is effectively occult to current imaging techniques.^5^^,^^6^ Positron emission tomography (PET) with amino acid radiotracers can demonstrate viable glioma cells beyond the enhancing lesion and is recommended where available for identifying biologically distant or metabolically active tumour sub-regions.^7^ However, its use is limited, particularly in the detection of early-stage infiltrative disease, due to its relatively low spatial resolution, still limited sensitivity and specificity, high cost, and logistic and accessibility challenges. There is a pressing unmet clinical need, therefore, to develop an imaging biomarker (IB) of infiltration in GBM for informing prognosis and first line treatment planning.

The recently proposed roadmap for the development and translation of IB for oncology^8^ identified three critical axes for successful biomarker development: (a) technical assay validation of the proposed metric, (b) biological/clinical validation of the underlying mechanism, and (c) IB translation cost-effectiveness. In IB development, the use of imaging methods already validated in multicentre studies and widely used in the clinic provides an advantage analogous to drug repurposing. Hence, while IB might be achievable using novel MRI or PET techniques, it would be beneficial to determine whether existing imaging modalities could be leveraged either to detect micro-invasive disease directly or measure a proxy for infiltration from the macroscopic tumour phenotype. The US National Cancer Institute (NCI) has launched the Co-clinical Imaging Research Resources Program (CIRP), which aims to optimize quantitative imaging methods for precision medicine and emphasizes data and software accessibility for patient-derived xenograft (PDX) models and biomarker generation.^9^

Diffusion weighted imaging (DWI) is the only potentially quantitative MRI approach which has entered clinical neuroimaging practice. The commonest quantitative measure derived from DWI is the apparent diffusion coefficient (ADC) and this has long been shown to relate to outcome in gliomas.^10^ As such, this has been widely studied and has undergone tissue validation and demonstration of robustness in several human and preclinical studies.^11^ Simulations and experimental studies *in vitro* suggest a negative correlation between ADC and tissue cellularity^12^ and clinical studies show that ADC tends to be lower in regions with higher cellularity.^13^ Two recent meta-analyses identified a significant negative correlation between ADC and cellularity in a range of tumours including gliomas.^14^^,^^15^

In this study, we proposed the rate of ADC change along macroscopic tumour borders as a biomarker for the infiltrating phenotype; however, validating the relationship between ADC and tumour cellularity is challenging due to limited sample size, spatial accuracy issues, ethical constraints for biopsy number and location, and millimetric misregistration between histology and imaging.^16^ While alternatives such as the combination of ADC and DWI images for detecting tumour infiltration,^17^ ADC tension coefficient (ATC) for the differentiation of 1p/19q co-deleted and non-co-deleted glioma,^18^ or postmortem histology, and machine learning hold promise,^19^ limitations such as tissue deformation and time lag between imaging and post mortem tissue retrieval remain a translational gap.

Preclinical studies combined with quantitative histopathologic assessment techniques complement this validation gap, providing spatially resolved mechanistic validation of IB with no delay between in vivo imaging and tissue data acquisition. We recently introduced a new quantitative histopathologic assessment technique called stacked in-plane histology (SIH), stacking several normalized signal histology sections prospectively cut in the MRI plane with image guidance to achieve high quality MRI/histology registration.^20^ SIH was applied to an orthotopic PDX mouse model of GBM, creating registered multi-dimensional datasets of MR images and histology. Immunohistochemical analysis of Human Leukocyte Antigen (HLA) expressed exclusively on tumour cell membranes enabled voxel-wise assessment of various MRI modalities against density maps of the patient derived human tumour cells within the mouse brain without interference from murine cells.

In this work, we propose to use the ADC slope beyond macroscopic core tumour margin, or marginal diffusion slope (MDS), as our IB metric and introduce a vector field approach to extend the sampling to characterize the whole of the tumour margin given the centrifugal radial pattern of infiltration observed immediately adjacent to the tumour (Figure 1). Disease progression is predominantly driven by this infiltrative phenotype, hence deriving our biomarker from the change in ADC at a position on the tumour margin where the dynamic range of values is sufficient to characterize an infiltrative phenotype could be predictive of outcome. Reflecting on the IB roadmap, this biomarker based on the MDS metric was developed in four synergistic steps: (a) Validation of the diffusion/tumour infiltration relationship in a clinically-relevant preclinical GBM model. (b) Assessment of the MDS IB in silico using a diffusion-infiltration theoretical model. (c) Quantitative histopathologic assessment of the relationship between the MDS IB and marginal tumour cell density to inform (d) an exploration of mean MDS as an IB predicting clinical outcome prior to consideration for multicentre external validation.

**Figure 1.**
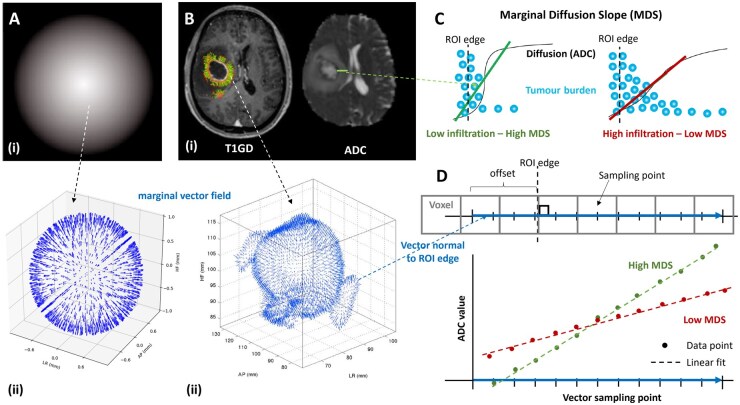
(A) Generating 3D surface vectors: (i) Phantom diffusion sphere with a negative radial gradient and (ii) distributed normal vectors on an arbitrary ROI surface co-centred with the phantom sphere. (B) MDS, measuring the apparent diffusion coefficient slope at the tumour core margins: (i) Enhancing T1w (CE) image with overlay of the vector field in the image plane (left) and corresponding ADC image (right). (ii) Example vector field of vectors normal to the enhancing T1w region. (C) Schematic of the marginal diffusion slope imaging biomarker (MDS IB) hypothesis mechanism: more infiltrated margins will lead to lower MDS than less infiltrative ones presenting more clearly defined edges. Note that not all infiltrating cells have to be detected. (D) MDS IB metric: Vectors normal to the ROI surface are sampled and interpolated ADC values for each sampling point are calculated. The marginal diffusion slope (MDS) for each vector of the marginal vector field is given by the slope of a linear fit to the curve of ADC values against vector sampling points converted to per mm based on the sampling frequency.

## Materials and Methods

### Study Design

This study, aiming at the biological validation and clinical exploration of a biomarker based on MDS along vector field normal to the tumour core, was designed following the framework introduced in the IB roadmap paper.^20^ It comprises four synergistic steps:

1. Assessment of MDS in a simplified diffusion-infiltration theoretical model.
2. Validation the ADC-infiltration relation identified by human sample meta-analysis studies of the literature in a clinically-relevant preclinical GBM model.
3. Quantitative histopathologic assessment of the relation between the MDS IB and tumour infiltration using tumour cell density maps (TCD) produced with the SIH method
4. Clinical assessment of MDS as an IB predicting overall patient survival in a pilot single centre cohort.

### MDS—Vector Field Analysis Using a Diffusion-Cellularity Model

The technique used for the 2D–3D vectorial analysis by tumour contour coverage is based on a previously introduced technique.^21^ Following the definition of the tumour extent by manual segmentation of the ADC maps, the first step is to refine the regions of interest (ROI) using an adaptive shape model. Then, the ROI edge voxels are extracted to define the macroscopic tissue boundary. The local curve topology at each surface voxel is sampled by a fixed kernel (5x5 voxels) and is defined by determining the solutions for the two principal eigenvectors: the tangential and the normal. Parametric map measures are then sampled at regular points along each normal vector centrifugally, as specified across a distance from inside to outside the macroscopic tumour margin at defined intervals. This novel sampling paradigm was analytically validated^21^ in a software phantom (developed in MATLAB) consisting of a sphere with a unit radial gradient. A spherical arbitrary tumour was used to generate normal vectors on this surface, allowing to measure signal along normal profiles (Figure 1A). To assess the ability of the vectorial approach to probe the diffusion-cellularity relation, a simplified model based on a 2D gaussian tumour cell density distribution was used to produce 2D diffusion maps using Graham’s diffusion equation at each voxel (D∝1/√ρ). The resulting diffusion maps were normalized to plateau at common brain parenchymal diffusion rates (8 × 10^−4^mm^2^/s) while a maximum cellularity threshold was introduced to simulate the tumour core. The infiltrative edges of these cellularity models were controlled by altering the standard deviation of the Gaussian diffusion front. ROIs of linearly decreasing diffusion steps were selected across the diffusion decay region and vector field normal to their surface generated. This permits simultaneous measurement of diffusion and cellularity along the vectors. MATLAB code for these simulations is accessible at https://github.com/UoE-ENTIRe/MDS-biomarker.

### Histopathologic MDS IB Assessment in a Preclinical Glioblastoma Model

Here, ADC assessment was performed using 3D datasets of registered MRI with SIH maps of HLA density, probing the patient derived human cells. To avoid unnecessary animal use, this work is based on data used for the validation of the SIH method. An overview of the MRI acquisition and methodology leading to tumour cell density (TCD) maps is ­proposed below; for further details on tumour modelling, MRI acquisition, histology preparation, and image registration one may refer to the original SIH publication.^20^ Briefly, G7 glioblastoma cells (10^5^ cells per mouse) were injected intracranially into CD1 nude mice (*n* = 10, one did not exhibit tumour growth). The animals were imaged with T2-weighted (T2w) and diffusion-weighted sequences using a Bruker-7T MRI system. Subsequently, brains were frozen, sectioned, labelled with HLA immunostaining, and scanned with a confocal microscope. The resulting histology images were registered with the MRI data using the SIH approach, based on mutual information, to generate TCD maps. ROI segmentation and data analysis were also performed as described in the original study.^21^

Statistical analysis: Two tailed student t-test was used for comparisons between MRI tumour regions and histology tumour regions. Spearman’s rank test was used to assess ADC-TCD correlation along vectorial profiles. Statistical ­significance flags: *P < .05, **P < .01, ***P < .001, ****P < .0001 and not statistically significant (NS.)

### Clinical MDS IB Comparison with Outcome

To determine the potential clinical value of the MDS as a prognostic biomarker for disease outcome, we conducted a single centre prospective clinical study as suggested in the IBR. In this phase, MDS was proposed as a predictor of survival as per the relationship to intervention. Specifically, a large MDS (steep slope of ADC spatial change) was hypothesised as representing a narrow transition between tumour and adjacent brain and therefore a less infiltrative phenotype which would be associated with longer survival. The IB was assessed using a Cox analysis against survival with the latter being at higher risk for pseudo-progression related errors (Figure S1).

Due to the challenges in obtaining a double baseline as recommended by IBR, a healthy volunteer study was conducted to measure the repeatability of ADC as measured in the same scanner and DWI sequence in normal brain. Due to the lack of suitable sampling for tissue validation for such a spatially specific and extensive measurement (perpendicular to the tumour margin across most of the tumour surface), biological validation relies on the computational and preclinical studies above to provide the necessary metric and tissue validation data to support the clinical MDS IB validation. The clinical study (patient and healthy volunteer) was approved by the regional research ethics committee. Informed consent was obtained from all participants.

#### Glioma Patients

Between October 2009 and September 2010, recruitment of patients with suspected HGG high-grade glioma (HGG) who were intended for safe maximal resection followed by temozolomide chemoradiotherapy was undertaken through the weekly tumour board multidisciplinary team meeting at the Greater Manchester Neurosciences Centre (Salford Royal Hospital, Salford, UK). While the recruitment was serial, not all patients were approached due to meeting clear inclusion or exclusion criteria or on the recommendation of the neurosurgeon, and some patients declined. Thirty patients were suitable for inclusion and were subsequently diagnosed with high-grade glioma (HGG) according to WHO 2007 criteria, and all commenced standard-of-care treatment (Stupp regimen) following maximal safe resection. All were commenced on 16 mg oral dexamethasone daily pre-operatively. Of these, 18 were male and 12 were female; mean age at diagnosis was 54 years 3 months (range 27 years 4 months–71 years 7 months). Six patients had non-glioblastoma diagnoses (three anaplastic astrocytoma and three gliomatosis cerebri) and were excluded. Of the remaining 24 glioblastoma cases, three were considered secondary glioblastoma (now classified as IDH-mutant) and one was subsequently reclassified as anaplastic oligodendroglioma based on 1p/19q co-deletion; these four were excluded from the glioblastoma analysis cohort. One glioblastoma patient died of pulmonary embolus and one was excluded due to HIV-positive status and associated medical co-morbidity. Eighteen patients were therefore eligible for MDS analysis. No patients were excluded due to imaging quality. The study was continued until all patients were deceased as assessed through follow-up in the Christie Hospital, Manchester or through the electronic patient record at Salford Royal Hospital, and the cause of death was examined for ­potential confounds. As a prognostic biomarker study, it is reported in accordance with the REMARK recommendations.^22^

#### Image Acquisition

All patients were imaged pre-operatively at 3 T on a Philips Achieva platform (Philips, Best, The Netherlands). In addition to standard clinical sequences (T1w with and without intravenous gadolinium-based contrast agent, T2w, and FLAIR) multiparametric imaging was undertaken using the Manchester brain tumour imaging protocol as part of a wider IB project.^21^ This included the Philips product ‘Medium DTI’ protocol comprising 16 non-colinear, non-coplanar diffusion directions at b = 800 s/mm^2^ and a single b = 0 s/mm^2^ acquisition at 2 mm isotropic spatial resolution covering the whole brain. ADC maps were generated from the standard Philips post processing software following linear coregistration. While more b = 0 s/mm^2^ acquisitions would be favourable, we utilised the product sequence and measurement stability is addressed separately by the healthy volunteer study (see Figure S2). No DWI distortion correction was used. The quality of coregistration between DWI and anatomical sequences was assessed on a case-wise basis by expert neuroradiologists; no cases had significant susceptibility distortion involving the tumour, and accurate coregistration of tumour was prioritised over other anatomical regions such as the frontal lobes.

##### Image Processing and MDS Generation

**Figure 2.**
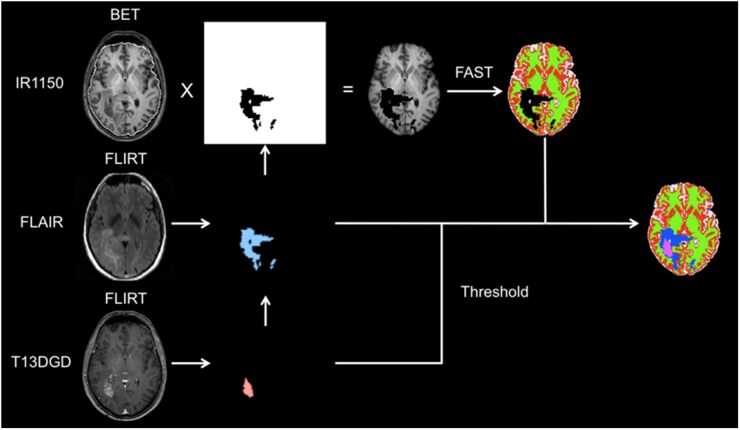
Segmentation approach for clinical data. FLAIR and pre-contrast T1w images were coregistered to the 1 mm isotropic post-contrast T1w image used for surgical planning. The whole FLAIR abnormalities and the enhancing abnormalities were manually segmented on each axial slice and then edited in the coronal and sagittal planes. These were then combined to create a mask covering the whole tumour abnormality. Non-enhancing core was defined as any region completely enclosed by enhancement. Normal appearing brain was segmented using FSL FAST using 3 classes on the FSL BET extracted brain tissue and combined to produce a 6-class segmentation for the purposes of measuring enhancing tumour margin profiles.

Images were exported as DICOM and converted to NIfTI for segmentation and analysis using dcm2nii. FLAIR and non-enhanced T1w images were linearly coregistered and resampled to match the 1 mm isotropic contrast-enhanced T1w image. Tumours were manually segmented using ITK-SNAP 3.0.0 into the following ROIs by GT and verified by two expert neuroradiologists (A.J. and S.J.M.): enhancing tumour, non-enhancing cystic/necrotic core, non-enhancing FLAIR abnormality. This combined ROI was ­suppressed, and the normal appearing brain was extracted and segmented into GM, WM, and CSF using FSL.^23^^,^^24^ An example of this is shown in Figure 2. ADC maps were coregistered and resampled to the T1w structural image used for segmentation.

MDS generation protocol and parameters are highlighted in Figure 1B-C. Tumour profiles were calculated from the normal of the external surface of the enhancing ROI and measured centrifugally from the enhancing tumour edge over 8 mm from 2 mm inside with 2 measurements per mm. In the case of multifocal or multicentric disease, all enhancing foci were included for measurement. Profile samples were valid only if the sampling was within the FLAIR abnormal volume of interest and more than 4 mm could be measured contiguously. This was to capture the profiles which could be robustly classed as bulk tumour with potential infiltration into surrounding abnormal brain, avoiding noise resulting from tumour infiltration being affected by anatomical boundaries such as pial or ependymal surfaces and to ensure ADC data were provided by more than 2 native ­contiguous voxels. The surface normal calculation and sampling code were written in house using MATLAB 2021 and is accessible at https://github.com/UoE-ENTIRe/MDS-biomarker. Importantly, the same code was used for the 3D clinical and 2D rodent MDS studies. ADC was sampled along each surface-normal profile from inside to outside the enhancing margin over a fixed distance window (−2 mm to +6 mm relative to the enhancing boundary; total 8 mm), at 2 samples per mm (sampling interval 0.5 mm). For each profile, MDS was defined as the slope from an ordinary least-squares linear regression of ADC versus distance (mm), yielding units of (mm^2^/s)/mm (with distance increasing centrifugally from tumour to peritumoural tissue). With regression implemented against the sample index, the resulting slope was converted to a per-mm value by multiplying by the sampling frequency (2 points/mm). The patient-level MDS was the mean of all valid profile slopes. Since the clinical outcomes could not be known at the time of image segmentation, the MDS measurements are *de facto* blinded to the outcome measure.

#### Statistical Analysis

As per the IBR and REMARK recommendations, the correlation of the predictor MDS against OS was calculated using Pearson’s moment. Given the relatively small clinical cohort, Cox’s proportional hazard’s model was undertaken on the MDS including age as a separate predictor given the homogeneity of the cohort. Analysis was performed using SPSS-22 and Cox proportional hazards models were fitted in R (v4.5.1) using the survival package (coxph), and the proportional hazards assumption was assessed using Schoenfeld residuals (cox.zph). There were no missing values for MDS or OS, and no censoring since the cohort was followed-up until all patients had died.

##### Repeatability Study on Healthy Volunteers

Due to the recruitment constraints from desired turnaround times from imaging to surgery, it was not possible in this study to undertake double baseline characterisation of the IB. This would have been desirable as recommended by the IB roadmap. In lieu of this, a repeatability study was undertaken using the same scanner and sequences on 15 healthy volunteers. Further details are included in the supplementary methods section (Figure S2).

DWI acquisition and ADC map generation was matched with the clinical study in glioma patients. To test repeatability, brains of the healthy volunteers were extracted using FSL BET and segmented into global grey (GM) and white matter (WM) using FSL FAST. Reliability of the ADC in healthy individuals for global GM and global WM was quantified by the intraclass correlation coefficient, Bland-Altman analysis, and the repeated measures coefficient of variation (RMCoV) as defined by:


(equation 1) $$\mathrm{RMCoV}=\frac{\mathrm{sd}(x_{1i}-x_{2i})}{(\sum_{i=1}^{n}\;\;\frac{x_{1i}+x_{2i}}{2})/n}$$

Using ANOVA, the ADC values from global, normal appearing GM and WM were also compared between the tumour patients and the healthy volunteers using a *P* < .05 to denote significant difference between values. Comparing global GM and global WM with normal appearing equivalent tissues in tumour patients, ANOVA reveals no significant difference between the healthy volunteers and tumour patients.

## Results

### Voxel Wise Analysis of Diffusion against Cellularity in the Perifocal Regions

**Figure 3.**
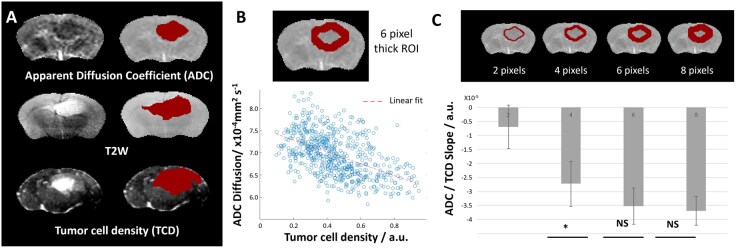
Voxelwise comparison of ADC against tumour cellularity in the region of interest to determine the dynamic range of the technique: (A) ADC, T2w, TCD maps and ROIs in an orthotopic G7 GBM model. (B) Perifocal ROI (thickness: 6 pixels) and corresponding ADC against cellularity plots with linear fit. (C) Increasing thickness ROIs at the ADC ROI margin, with the corresponding slope of the linear fit to the ADC against cellularity data.

The diffusion/cellularity relationship was first assessed using preclinical data with accurately spatially- matched histopathology data. Previous work has demonstrated the robustness of ADC measures in preclinical scanners and comparability to human measurements.^10^ The orthotopic xenograft G7 GBM model used for these experiments was chosen as it has been shown to exhibit spatiotemporally predictable histological features of human disease, including a dense core, central necrosis, and infiltrative tumour margins.^25^  Figure 3A shows ADC and T2w images of the G7 GBM model, with the corresponding TCD maps (HLA binding representing tumour cell density). Manually selected ROIs highlight tumour-related abnormal regions, with inter-observer ADC and T2w segmentation variance for this model typically below 20%.^25^ As in the simulations shown above, ROIs of increasing thickness were placed around the ADC ROI and a voxelwisevoxel wise comparison between ADC coefficient (diffusion) and the normalized tumour cell density of the TCD map was performed (Figure 3B). The resulting curve shows a similar relationship to previously published clinical data of ADC against biopsy cellularity in individual patients^5^ (Figure S3). For ROI thickness between 4 and 8 voxels a strong negative correlation was observed between ADC values and tumour cell density (Figure 3C).

### Histopathologic Validation of MDS as a Biomarker of Glioblastoma Cell Infiltration

After confirming the presence of a negative relation between ADC and tumour cellularity in our rodent data, we focused on the MDS IB biological validation. An assessment of our surface-normal vectorial analysis code was first performed on a simple model reproducing the negative slope between diffusion and cellularity and allowing to simulate more infiltrative fronts by increasing the standard deviation of the Gaussian cellularity distribution (Figure S4). As expected, the in-silico results showed a negative relation between the ADC slope and infiltration beyond the core regions, with more infiltrative margins leading to lower ADC slope. Furthermore, this modelling paradigm allowed to explore the dynamic range sensitivity of the technique, that was crucial for optimizing MDS performance on the rodent data.

**Figure 4.**
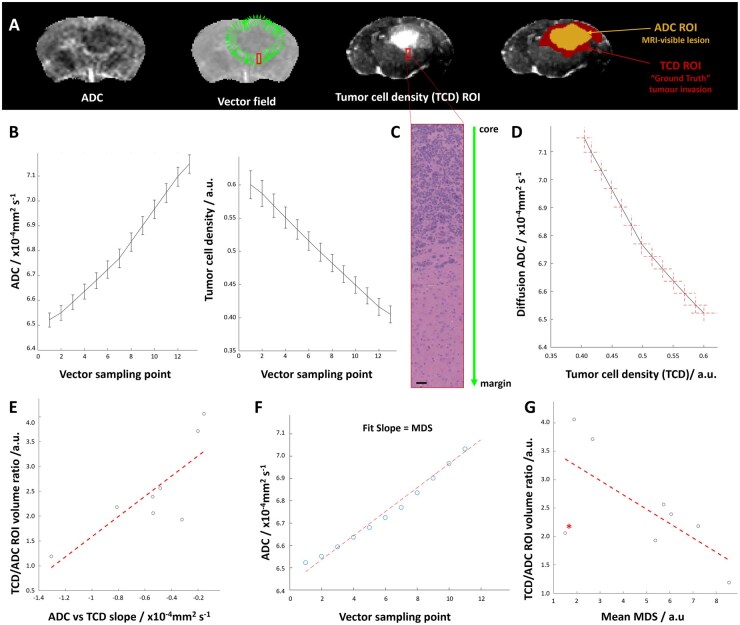
A quantitative relationship between ADC slope and tumour cellularity: (A) ADC, Vector profiles normal to the ADC ROI, tumour cell density map (TCD) and ADC/TCD ROI comparison. (B) Average diffusion and tumour cell density along the profiles. (C) H&E images at increasing resolutions across the tumour core margin highlighted in A. (D) Diffusion against tumour cell density plot for the data shown in A-B. (E) TCD/ADC volume ratio against ADC vs TCD slope (*n* = 8). (F) MDS as the fit of the ADC against vector profile points for the data shown in A. (G) TCD/ADC volume ratio against MDS the average MDS across all vector profiles (*n* = 8). The starred point represents the mouse in which several vectors exceeded the brain regions, partially compromising the robustness of the analysis (Figure S6).

Figure 4A shows an ADC image and the corresponding normal vector distribution around the manually-delimited ROI. Diffusion (ADC) along the vector profiles is shown to increase with distance from the macroscopic tumour margins while tumour cell density (TCD) decreases, a trend also visible in H&E images of the same region (Figure 4B-C). A strong negative correlation was observed between diffusion and TCD maps (Figure 4D). This correlation was systematic for each of the studied mice (Figure S5). One mouse where the size and position of the tumour caused several vectors to exceed the brain regions exhibited a lower correlation (Figure S6). Also, for one mouse dataset that had to be removed because the Human Leucocyte Antigen (HLA) stained tissue was damaged, replacing HLA by H&E images we were able to reproduce the strong negative correlation between ADC and tumour infiltration observed in the simulations (Figure S7).

The ratio of the TCD to the ADC ROI gives information about the importance of non-probed tumour infiltration, with higher ROI_TCD_/ROI_ADC_ corresponding to more infiltrative cases. The preclinical data showed a strong correlation between TCD/ADC and the ADCvsTCD slope (Figure 4E). Here, as for the model, a stronger negative marginal ADC slope corresponded to a less infiltrative tumour margin on spatially-matched histopathology. Finally, to evaluate the ability of the MDS vectorial approach to provide a robust infiltration biomarker, an analysis of ADC slope alone was performed by studying average ADC change along the vector profiles (Figure 4F). Figure 4G reveals a strong correlation between the mean MDS and tumour infiltration measured as ROI_TCD_/ROI_ADC_. Similar results were observed when comparing mean MDS to the ROI_TCD_/ROI_T2w_ ratio, an additional indirect marker of marginal invasion (Figure S8B).

### Spatially Resolved Assessment of the Relation between Diffusion and Glioblastoma Cellularity

**Figure 5.**
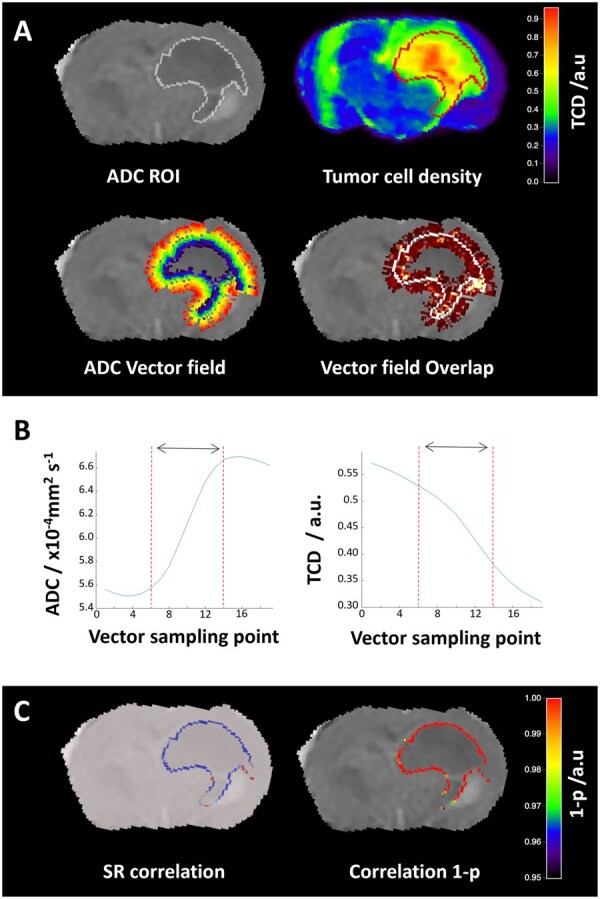
Spatial correlation between diffusion and tumour cellularity: (A) Manually defined ADC ROI on ADC and TCD maps. The artefact on the left hemisphere of the TCD map corresponds to folding in one of the stacked sections (included as it was not affecting the region of interest). The MDS vector field is formed normal to the ADC ROI centrifugally (vector profiles going from blue to red) and a vector field overlap map allows to identify regions with significant vector overlap or multiple sampling which are minimal. Note that the cellular infiltration on the TCD map extends well beyond the ADC macroscopic tumour boundary in several locations which are not limited by anatomical boundaries such as neocortical interfaces or ependyma. (B) example interpolated ADC and TCD data along a vectorial profile. Note the similarity to the choice of gaussian for the in silico experiments. The dynamic ADC range along this profile, where optimal MDS analysis is expected, is highlighted with a black arrow (C) Spearman’s rank (SR) correlation of ADC against TCD showing from high negative (blue) to high positive (red), and 1- p of the SR analysis for each vectorial direction. Histological inspection of regions of without significant correlation reveal natural anatomical boundaries to tumour cell infiltration.

While the average ADC slope was assessed above, it is important to understand that the proposed method produces MDS measurements along each surface vectorial direction, potentially resulting in a spatially-resolved biomarker of infiltration (Figure 5A). In the preclinical setting, this could be assessed by the ADC-TCD correlation along each profile (Figure 5B). The negative correlation observed globally is here spatially-localized and reproduced for most margins of this ROI with high significance (cf. Figure 5C, 1-p map). The resulting correlation maps, not only strengthen the overall IB results, but highlight how predicted infiltration could be spatially visualized, extending the MDS IB utility beyond a summary metric and overall prognostication into a spatially-resolved surface map of infiltration.

### Clinical MDS Slope IB Relates to Outcome

**Figure 6.**
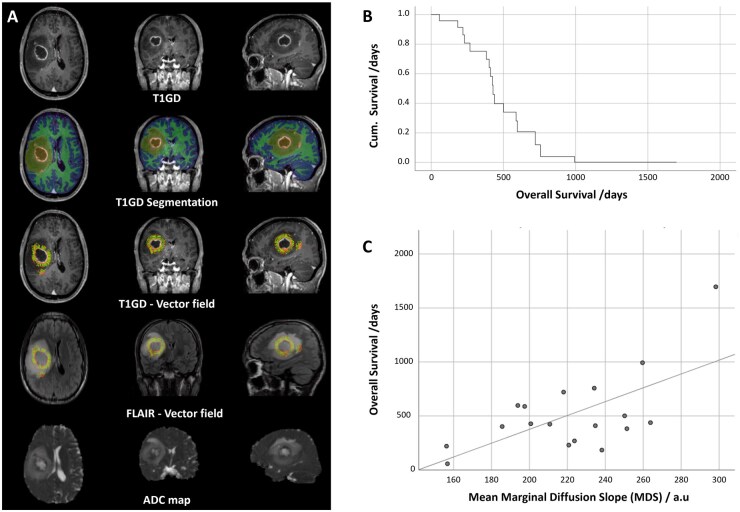
Clinical MDS IB assessment: (A) Representative slices centred through an example GBM, with: post contrast T1-weighted 3D FFE image (T1GD); tissue segmentation overlay; surface normal trajectories used to sample the ADC values showing the transition from the enhancing tumour boundary (orange) through the non-enhancing FLAIR abnormality; same trajectories are overlaid to FLAIR images and finally the linearly rigidly coregistered ADC maps using FSL FLIRT that were used for determining MDS along the profiles. (B) Survival function and (C) scatterplot of OS in days against mean MDS for each patient (*n* = 18).

The relationship between MDS and outcome was assessed in a group of 18 patients. In the analysed cohort (*n* = 18), median overall survival (OS) was 426 days (14.0 months), with a 95% confidence interval for the median of 382–721 days (12.5–23.7 months). Mean OS was 516.5 days (17.0 months), and OS ranged from 57 to 1696 days (1.9–55.7 months). Figure 6A shows an example dataset with the corresponding segmentation and the vector field normal to the enhancing tumour edges, extending into the FLAIR hyperintense region surrounding it. The time taken for manual segmentation was less than 15 minutes per patient and MDS was successfully measured along the vectorial profiles using registered ADC maps. Of note, this segmentation follows the Multimodal Brain Tumour Image Segmentation Benchmark (BraTS) procedure.^26^ The survival function and a scatterplot of OS against MDS is shown in Figure 6B-C. This scatterplot demonstrates a positive linear correlation, with higher MDS (i.e., a steeper increase in ADC from inside to outside the tumour margin) associated with longer survival. Pearson’s correlation between MDS and OS was 0.636 (*P* < .005). Cox proportional hazards regression (see Figure S9) showed that MDS was significantly associated with OS (model *P* = .013; HR per 1 SD increase in MDS = 0.47, 95% CI 0.25–0.89). In a multivariable model including age (see Figure S9), MDS remained significant (*P* = .010) while age was not (*P* = .35). Proportional hazards assumptions were supported by Schoenfeld residual testing (*P* = .76; Figure S9).

## Discussion

### MDS is a Biomarker Predicting OS

Marginal tumour infiltration beyond the contrast-enhancing borders is the major cause of GBM recurrence and treatment resistance. The clinical component of this translational study in GBM patients demonstrates that the mean change in ADC values across the margin of the tumour on preoperative imaging (i.e., MDS) is predictive of OS in this small single centre cohort. This supports the *a priori* hypothesis that if ADC is a proxy for tumour cellularity, then a more abrupt change in marginal ADC values indicates a less infiltrative phenotype with fewer cells beyond the macroscopic margin and *vice versa* (Figure 1C).

### Following the Roadmap to Novel Biomarker Validation

Given the unmet need to determine marginal infiltration at the GBM margin to improve prognostication and image-guided therapy, this represents a promising biomarker candidate. However, recent scrutiny has rightly emerged concerning the development and validation of IBs in oncology, with a landmark roadmap paper forming the framework for successful translation published in 2016. Before a biomarker such as MDS could be meaningfully progressed, in accordance with the IB roadmap, further technical and biological validation work would be necessary.

Here we have presented an exemplar of how such a process can be approached. By implementing the MDS vector field analysis on a simple model of cellular density against diffusion we were able to assess the robustness of both our infiltration model and practical MDS implementation. The proposed biomarker was based on ADC, one of the most widely available and robust MRI measures, showing repeatable measurements on commercially available calibration instruments across sites and scanner types.^27^ Additional prevention to quantitation uncertainties was provided by considering the diffusion slope instead of the actual diffusion value. Given the challenges associated with tissue validation of MDS in humans (limited sampling with significant location bias, spatial error, and large-scale difference between tissue and voxel size), it was necessary to undertake preclinical experiments in a rodent model, where these challenges could be directly addressed. This required the testing of assumptions made in the human IB concept (i.e., that ADC is predictive of GBM cellularity) to determine if they were recapitulated in the animal model. We therefore carried out tissue-imaging correlation experiments using the preclinical model and our recently developed SIH technique to address these challenges with a level of precision which would not be possible in humans. Using this approach, we have demonstrated that in an infiltrative (G7) human GBM rodent xenograft model, tumour cellularity is a significant contributor to the ADC values measured by DWI, and that reassuringly this relationship recapitulates the exponential relationship seen in human studies (Figure S3).

Having established that ADC is related to cellularity in both humans and rodent models, we measured MDS in the rodents using the same approach and software as in the clinical study. Since it is not currently possible to determine the true extent of infiltrating disease in patients, we determined an index of infiltration by taking the ratio of visible ADC abnormality to the footprint of infiltrating disease using immunohistochemistry which was beyond that directly evident on the ADC map, indicating that microinvasive disease extends beyond the ADC margin.

MDS in the rodents was negatively correlated with this ratio, indicating that it is a true summary measure of an infiltrative phenotype. This experiment would not have been practicable in humans given the limitations for supramarginal sampling at distance from the enhancing tumour and very limited potential for paired pre-mortem imaging with post-mortem whole tumour assessment, demonstrating the complementary role of preclinical model experiments for specific biomarker validation studies recommended by CIRP.

While amino acid PET imaging,^7^ particularly with ^18^F-FET, can demonstrate viable glioma cells beyond the enhancing lesion, MDS offers several practical advantages for clinical implementation. Diffusion MRI provides superior spatial resolution (2 mm isotropic) compared to clinical PET (4-5 mm), involves no ionising radiation exposure, and is available on all clinical MRI platforms rather than requiring specialist PET centres with cyclotron access for tracer production. Furthermore, MDS can be derived from standard clinical diffusion sequences with no additional acquisition time or cost, facilitating integration into existing pre-operative and pre-radiotherapy workflows. We envisage potential clinical applications including: (1) pre-surgical prognostication and informing extent of resection decisions, potentially in combination with intraoperative fluorescence guidance; (2) pre-radiotherapy target volume delineation to account for infiltrative disease beyond contrast enhancement; and (3) baseline characterisation of infiltration likelihood to aid interpretation of subsequent follow-up imaging. While MDS may lack the metabolic specificity of amino acid PET, its accessibility and ease of implementation provide a lower barrier to large-scale clinical validation and eventual translation.

Recent work has explored imaging-based prediction of glioblastoma infiltration and recurrence using radiomic features and machine learning, as collated in a recent comprehensive review.^28^ While these approaches show promise, they can be limited by the ground truth provided, and the underlying algorithms or biological basis can be uncertain or unknowable, creating their own validation challenges. By contrast, MDS is grounded in a testable biophysical hypothesis that can be validated through direct tissue correlation, as demonstrated in our preclinical experiments.

These approaches need not be mutually exclusive. AI-derived metrics could benefit from the preclinical MRI-histology validation mechanisms deployed here, or be combined such that algorithms are informed or weighted by mechanistically-grounded parametric measurements such as MDS. Recent work demonstrating that DCE-MRI-derived interstitial fluid flow vectors predict invasion patterns^29^ provides independent support for vector-based marginal analysis as a valid methodological framework. We have recently been awarded funding to test MDS alongside selected radiomic features in a multicentre clinical trial cohort, which will allow direct assessment of complementarity between mechanistic and data-driven approaches.

### Study Limitations

Rodent GBM models, while providing unique preclinical insights otherwise impossible, are not entirely representative of human disease. Differences include tumour scale, potential lack of human-like molecular heterogeneity, often absent adaptive immune systems, and less WM for infiltration. Direct comparability of imaging techniques between species can also be limited, and rodent models are not typically treated (e.g., corticosteroids). Importantly, potential biological contributors to the ADC gradient other than cellularity. Such as extracellular oedema, necrosis, or perfusion, might differ in their impact. Nevertheless, it is critical to mention that the G7 model presents all these biological contributions too: (a) an oedematous region (visible on T2w) expanding beyond the ADC ROI as for clinical GBM, (b) complex micro-necrosis patterns within the infiltration zones that tend to become sparser with distance from the injection point (Figure S10) and (c) perfusion dropping towards the non-neovascularised tumour injection point; potentially associated to vascular co-option/cuffing mechanisms also present clinically beyond the angiogenic active tumour regions.^25^ Nevertheless, by applying the SIH technique for accurate MRI-histology coregistration in this patient-derived model, we were able to show how spatial ADC changes predicted cellularity changes and confirm the ADC-cellularity relationship. For this preclinical study, our focus was on measuring the change in ADC across the macroscopically visible core tumour margin, rather than its relationship to enhancement, leading to a difference in delineation approach. Contrary to the clinical study, we were not blinded to ADC but remained blinded to cellularity while delineating macroscopic tumour on ADC maps and the full extent of macroscopic abnormality on T2w imaging.

For the clinical study, no double baseline measurements were acquired due to short intervals between diagnosis and surgery and patient recruitment challenges, common in high grade tumour studies. This was partially addressed by conducting a healthy volunteer study using the same scanning protocol (Figure S2), complementing existing DWI/ADC literature on repeatability in normal brain. Despite the small patient cohort, typical limitation of single-centre GBM studies, this study is an important step of the IB roadmap and a robust basis for multicentre deployment. Future multicentre studies, using WHO 2021 criteria and whole-genome sequencing, will also address the molecular marker limitations of this WHO 2007-based study and introduce a centralised RANO assessment for PFS. OS was chosen as the primary endpoint due to historical limitations in clinic-radiological progression diagnosis (i.e., pseudophenomena, MGMT promoter methylation status, and post-operative imaging within 72 hours not routinely available). Patient tissue for direct MDS validation, though preferable, was unrealistic, as the biomarker’s nature required en bloc tumour resection in marginal regions, with neurosurgical advice indicating up to 2 mm misregistration in targeted samples. Thus, our preclinical study was crucial for precise, contiguous margin assessment. The use of GBCA enhancement for tumour margin guidance, while the current standard for surgical resection and radiotherapy planning, has limitations due to the biological specificity of blood-brain barrier disruption but avoids potential biases related to manually segmenting and thus placing measurement profiles directly onto ADC maps. In the planned larger validation cohort, we will also evaluate segmentation-related reproducibility by testing boundary perturbations and inter-rater variability, leveraging several automated segmentation methods to quantify their impact on MDS. Finally, the MDS IB definition simplifies the complex tumoural interface. While biological reality suggests a sigmoidal profile, variability and noise made fitting such a function unstable. A simpler linear fit was chosen, which might overestimate infiltration where the linear component is narrow by lowering the apparent gradient due to exponential and asymptotic components. This would more likely lead to a Type II statistical error by reducing discrimination between well-circumscribed and highly infiltrating profiles, a risk deemed acceptable for robust biomarker measurement. Future works could focus on refining the sensitivity of the metric by the application of either modulable vector length based on spatial transition or the use of more complex fitting models.

In conclusion, we presented an exemplar of an IB development and validation approach which is compliant with the IB Roadmap while addressing the limitations frequently encountered in glioma imaging research. The MDS was hypothesized as a pre-operative IB of glioblastoma infiltration phenotype with prognostic implications. It has been shown in a small single centre prospective cohort clinical study to be predictive of OS. A pragmatic, prospective, multicentre study (Tessa Jowell BRAIN MATRIX^30^) is currently underway which will provide imaging, clinical, and molecular data along with residual enhancing disease, RANO progression free survival, and OS to determine the external validity, generalizability, and utility of the MDS as a prognostic biomarker. Furthermore, our results highlighted the spatially resolved nature of the relation between MDS and tumour invasion suggesting the potential use of MDS as a tool for assisting radiotherapy planning in regions not probed by current clinical imaging protocols. We overcame the limitations in characterizing and validating the biomarker in humans directly through tissue sampling, by employing computational and preclinical techniques to provide translational evidence for the validation roadmap, both as a go/no-go for a wider clinical study beyond translational gap 1 and to inform the design and interpretation of such a study. We insist on the novelty of this methodological approach and commend it to others looking to rationalise development of IBs in a setting of increasing demands on patients for therapeutic clinical trials.

## Supplementary Material

vdag028_Supplementary_Data

## Data Availability

Code for in vitro, preclinical, and clinical analysis was made public alongside preclinical datasets.
