# Supplementary material for: Marginal diffusion slope as a prognostic imaging biomarker of infiltrating phenotype in glioblastoma; A cancer imaging biomarker roadmap study
Source: Neurooncol Adv. 2026 Feb 17;8(1):vdag028. doi: 10.1093/noajnl/vdag028 (PMC13034536; doi:10.1093/noajnl/vdag028)

Supplemental information for: Marginal Diffusion Slope as a Prognostic Imaging Biomarker of Infiltrating Phenotype in Glioblastoma; A Cancer Imaging Biomarker Roadmap Study

Fig. S1. Overall survival (OS) versus progression-free survival (PFS)

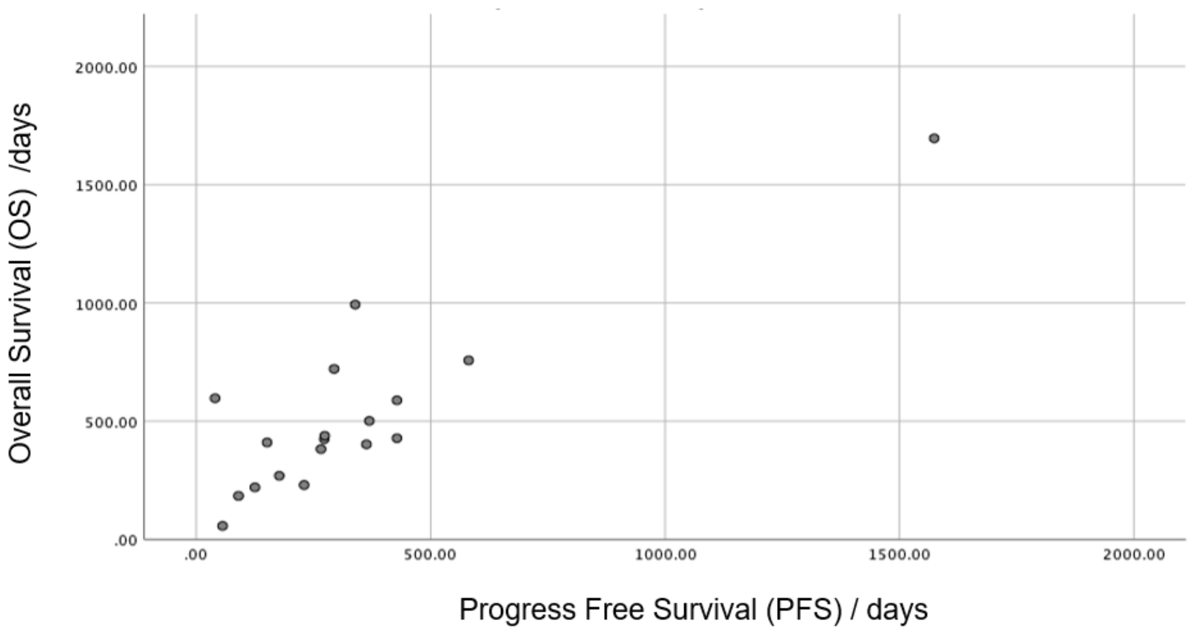

This plot shows that for most cases, there is a clear positive linear correlation between PFS and OS. There are 3 cases however, where the OS is much longer than would be predicted by the PFS (i.e. they are conspicuously above and to the left of the regression line). This may reflect pseudoprogression, interval-censoring, or assessment variability.

Fig. S2. Repeatability study on Healthy Volunteers

The standard institutional exclusion criteria for the healthy volunteer study are shown below:

- Standard MR exclusion criteria (inc. pregnancy)
- Age < 18 or > 49
- Previous neurosurgery or skull base surgery of any kind
- Previous non-research neuroimaging of any kind
- Previous lumbar puncture
- Any diagnosed neurological condition including:
  - o Migraine
  - o Chronic Headache
  - o Epilepsy
  - o Neoplasm
- Previous significant head injury

- Known or suspected autoimmune disorder, vasculitis or connective tissue disorder
- Personal or family history of polycystic kidneys
- Family history of intracranial haemorrhage
- Tremor or other movement disorder
- Current upper respiratory tract infection
- Medication with potential to affect cerebral blood flow, including intake of caffeinated drinks greater than 4 cups per day or headache resulting from caffeine abstinence

The mean age of participants was 29.2 years (range 21 – 48 years). Nine were female and 6 were male. Repeat imaging was undertaken at the same time of day for each participant to minimise diurnal effects. The median interval between visits was 14 days. Reliability of the DTI parameters in individuals for global GM and WM as quantified by Bland-Altman analysis and repeated measures coefficient of variation (RMCoV).

Parameter	Tissue	Δ Lilliefors Normality	Kendall's τ *p**	Lower 95% CI	Upper 95% CI	Percent. Lower 95% CI	Percent Upper 95% CI	*cov* %
DTI HR MD	GM	Y	0.4354	-1.53×10^{-5}	2.3×10^{-6}	-1.89%	2.83%	1.21%
DTI HR MD	WM	Y	0.59	-1.19×10^{-5}	1.19×10^{-6}	-1.68%	1.67%	0.93%

* Kendall's τ for correlation between differences and means to determine value-dependent heteroskedacity. With Bonferroni correction, none are significant.

RMCoV was <2% and the ICC >0.99 for all the DTI HR MD (ADC) measures in global GM and global WM. Bland-Altman 95% confidence limits of agreement were -15.3×10^{-6} to 23.0×10^{-6} mm^2/s for GM and -11.9×10^{-6} to 11.9×10^{-6} mm^2/s for WM. Comparing global GM and global WM with normal appearing equivalent tissues in tumour patients, ANOVA revealed no significant difference between the healthy volunteers and tumour patients.

Fig. S3. Clinical data analysis of mean ADC against tissue cellularity, and comparison with our mouse data

Supplementary material from Eidel et al. (cf. manuscript ref. (2)) was used for assessing the clinical relevance of the distribution of ADC against tumour cellularity. The scatterplot of mean ADC against mean cellularity from 37 patient datasets is shown below. Allowing for some expected differences (e.g. physiological scale, field strength), this clinical data plot presents a similar pattern to the distribution of ADC against tumour cellularity observed in our preclinical data (cf. Figure 2.B, reproduced below).

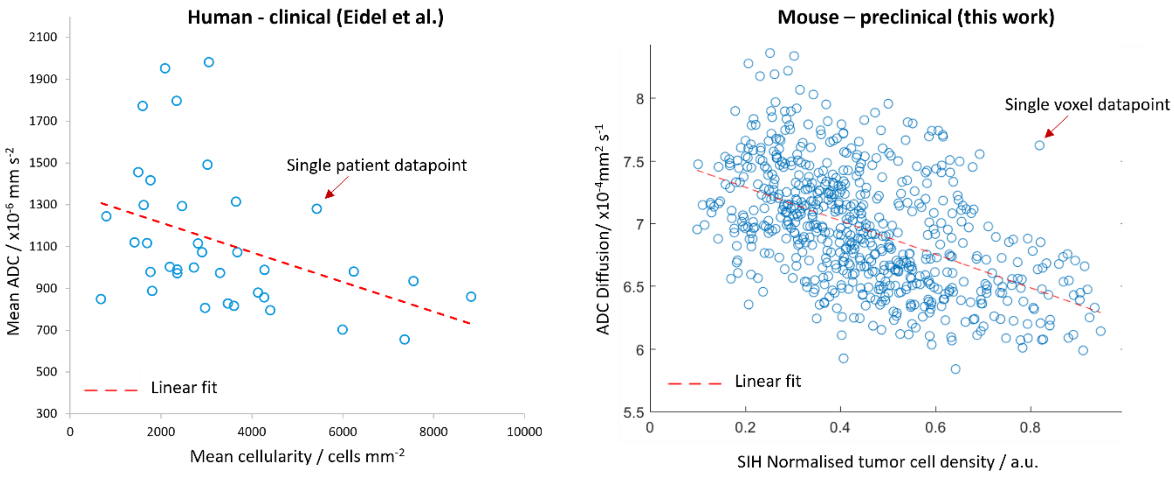

Fig. S4. A simplified cellularity-diffusion model probing the potential relationship between MDS and marginal tumour infiltration in silico

While spatio-temporal GBM infiltration models incorporating reaction (cell division) and diffusion (cell motility) commonly employ partial differential equations with a logistic reaction term (the Fisher equation), here we only require a single initial time point model to assess the baseline advancing tumor front state across the brain domain. For the remainder of this work, diffusion refers to water diffusion as measured using ADC from DWI and not cellular diffusion or motility. A model based on 2D Gaussian initial tumour cell density allowed production of synthetic water diffusion maps using Graham's diffusion-density equation (Fig. S4A). Profiles from the centre to the edge of the images allow visualization of the relationship between cell density and water diffusion in this model, with a proximal cellular density threshold defining the viable tumour core at maximum carrying capacity and a distal hypocellular limit for adjacent brain.

To assess the dynamic range of the relation between diffusion and density beyond an arbitrarily defined tumour core margin, contour ROIs of increasing thickness were placed at the viable core edge and plots of modelled water diffusion against cell density (Fig. S4B) reproduced the expected reduction in diffusion with increasing cell density based on prior work. Increasing ROI thickness led to a more robust negative slope on the diffusion/cellularity plot (Fig. S4C), increasing the dynamic range of the technique. By increasing the standard deviation of the Gaussian cellularity map as a proxy for higher reaction-diffusion it is possible to simulate more infiltrative fronts, without affecting the relationship between diffusion and cellularity (Fig. S4D). A vector field normal to the viable core edge allows probing of the negative relation between water diffusion and cellularity (Fig. S4E) across the margins, and assessment of the effect of infiltration on the mean MDS, the slope of diffusion along vector profiles normal to the core edge. Fig. S4F shows how the average diffusion range within the length of the normal fixed-length vector decreases as cellularity increases, leading to a steeper diffusion slope for less infiltrative fronts. The appropriateness of a linear fit increased with increasing standard deviation of the

cell density distribution, due to lower diffusion variation over the length of the vector profiles and closer approximation of the gaussian slope.

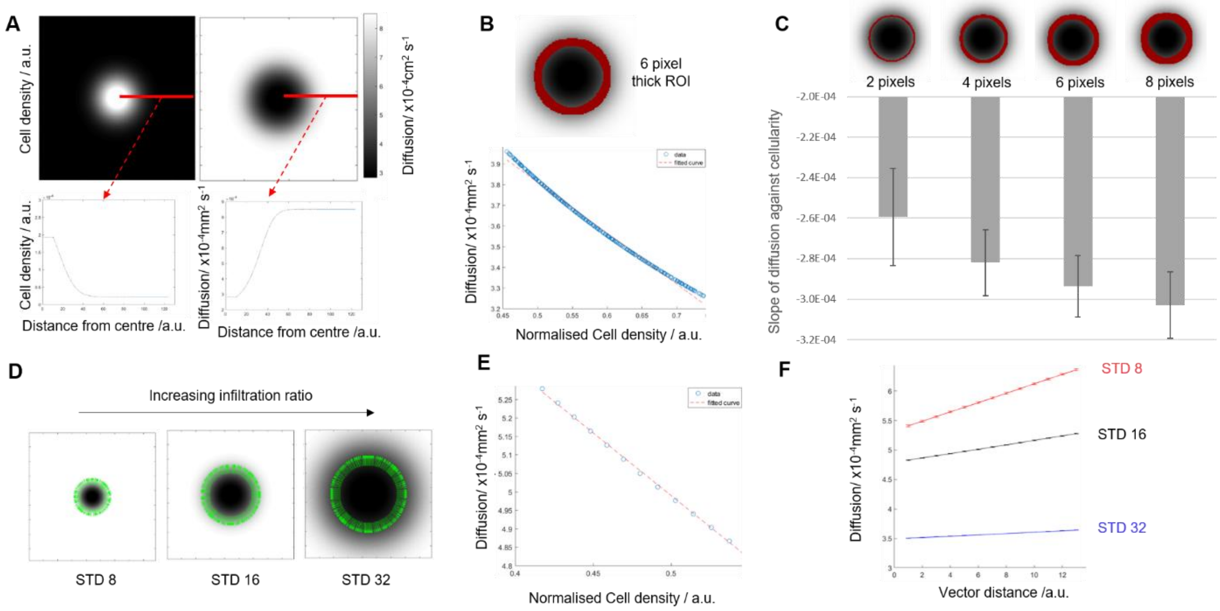

Modelling the relationship between cellularity and diffusion: (A) Simulated cell density and water diffusion maps and profiles from the centre to the edge of these maps. (B) Diffusion against cellular density plot for 6 voxel thick ROI covering the density decay region. (C) Slope of the water diffusion against cellular density curve for increasing ROI thickness. (D) A vectorial profile analysis of diffusion against cellularity: increasing infiltration diffusion maps produced by increasing the standard deviation of the modelled cell density (STD=8, 16, and 32). Each green arrow presents a centrifugal sampling profile. (E) Fit of the water diffusion against cell density data for STD16. (F) Diffusion against position along the vector profiles for increasingly infiltrative fronts (increasing STD). MDS, the slope of these plots, decreases for more infiltrative edges.

Fig. S5. Average ADC and HLA-SIH along vector profiles and ADC against HLA-SIH for all studied mice

Outliers: M02 is not present as it did not develop a tumour. For M07 tumour growth was rapid causing several vector points expanding beyond the brain not to be considered (see details in Fig. S2). M09 HLA staining was of unacceptable quality and H&E had to be considered as an alternative measures of tumour invasion (see details in Fig. S3).

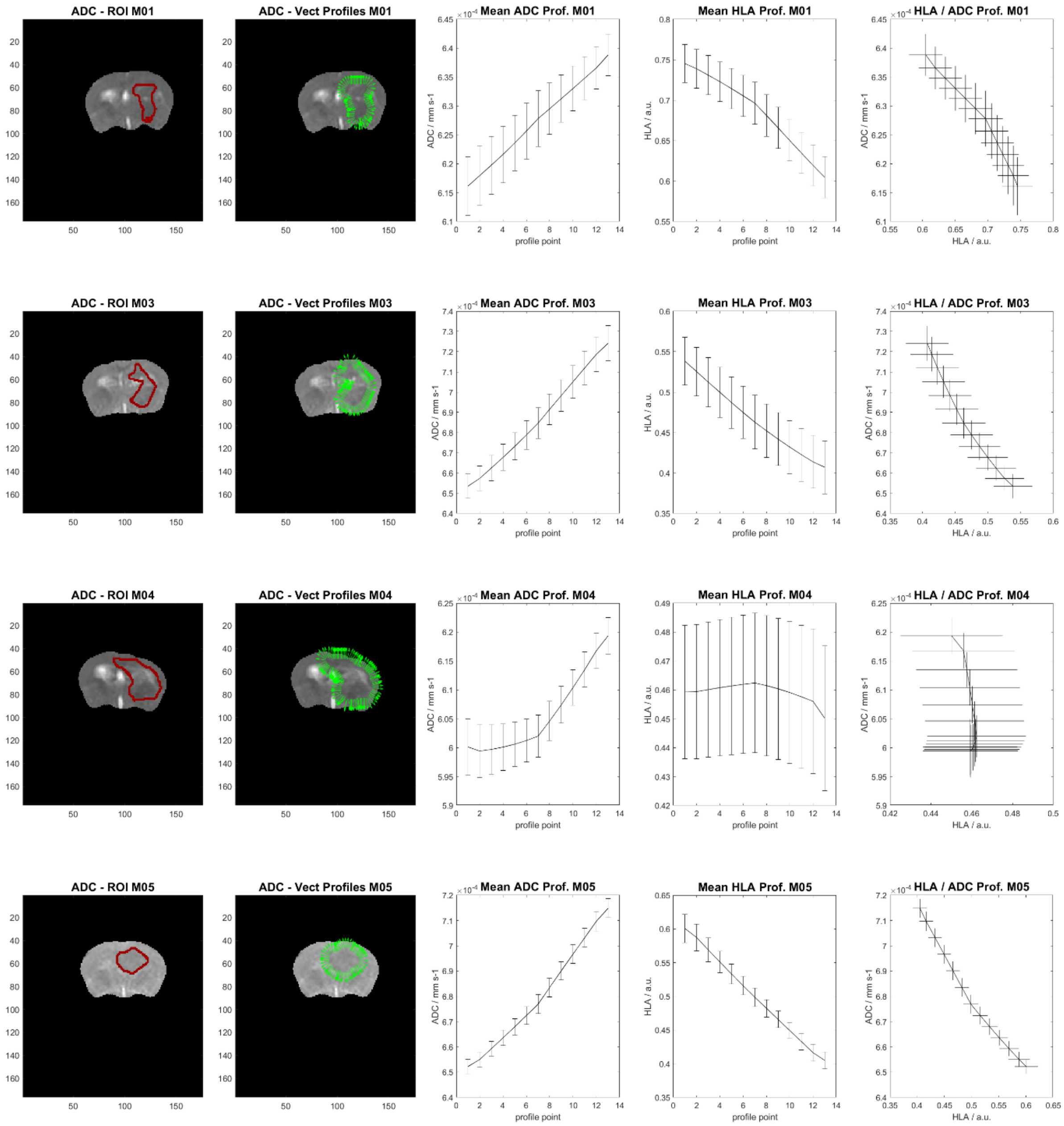

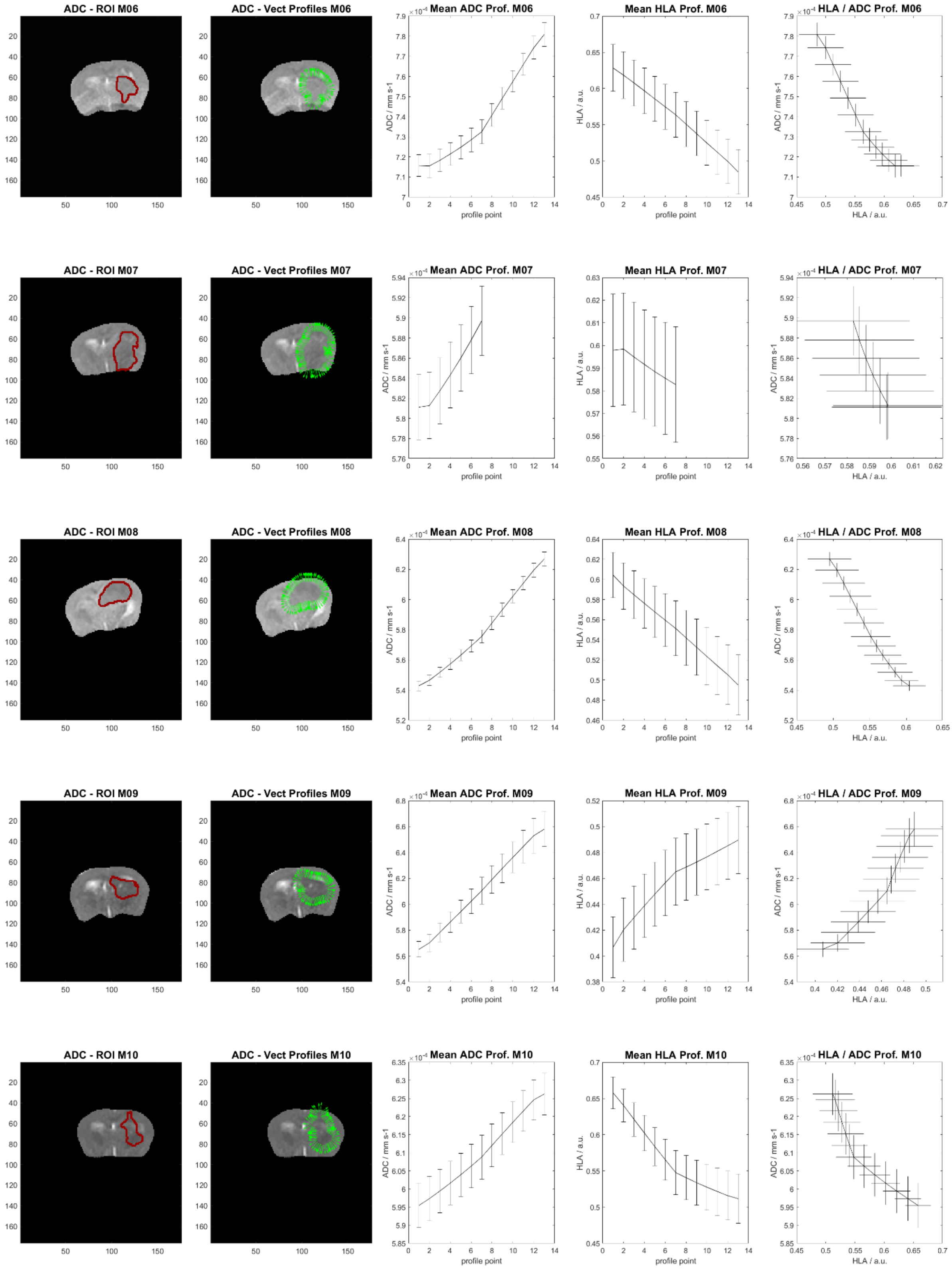

ADC - ROI M06
ADC - Vect Profiles M06
Mean ADC Prof. M06
Mean HLA Prof. M06
HLA / ADC Prof. M06
ADC - ROI M07
ADC - Vect Profiles M07
Mean ADC Prof. M07
Mean HLA Prof. M07
HLA / ADC Prof. M07
ADC - ROI M08
ADC - Vect Profiles M08
Mean ADC Prof. M08
Mean HLA Prof. M08
HLA / ADC Prof. M08
ADC - ROI M09
ADC - Vect Profiles M09
Mean ADC Prof. M09
Mean HLA Prof. M09
HLA / ADC Prof. M09
ADC - ROI M10
ADC - Vect Profiles M10
Mean ADC Prof. M10
Mean HLA Prof. M10
HLA / ADC Prof. M10
ADC / mm s-1
HLA / a.u.
profile point

Fig. S6. Case of MDS vector field sampling outside the brain (M07)

In the case of Mouse 7, the size and location of the tumour made that the normal vectors to the tumour-surface were often covering areas beyond the brain margin, not reaching sufficient length to populate and support a robust profile analysis with the same number of points as for other mice. Nevertheless, for the 8 points covered by most vectors, the expected negative relation between ADC and HLA was observed.

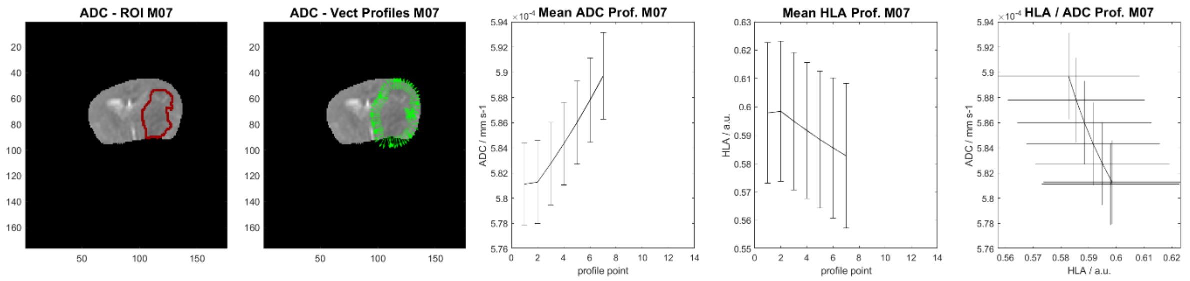

Fig. S7. MDS assessment with H&E-TCD in excluded mouse dataset due to low quality HLA-TCD (M09)

In Mouse 9, HLA immunostaining was of unexpectedly poor quality within the tumour core, with marked signal dropout. This likely reflects technical staining failure and/or effects of early necrotic core formation, and is visible as a substantial reduction in HLA signal relative to the corresponding H&E section(Fig.S7A). To retain this dataset for analysis, we therefore used H&E as the histological comparator for this case, as H&E staining appeared unaffected by the HLA immunohistochemistry abnormality. Using H&E-derived tumour cell signal intensity (Fig.S7B), the ADC profile demonstrated the expected negative relationship (i.e., higher cellular signal associated with lower ADC), consistent with our working hypothesis.

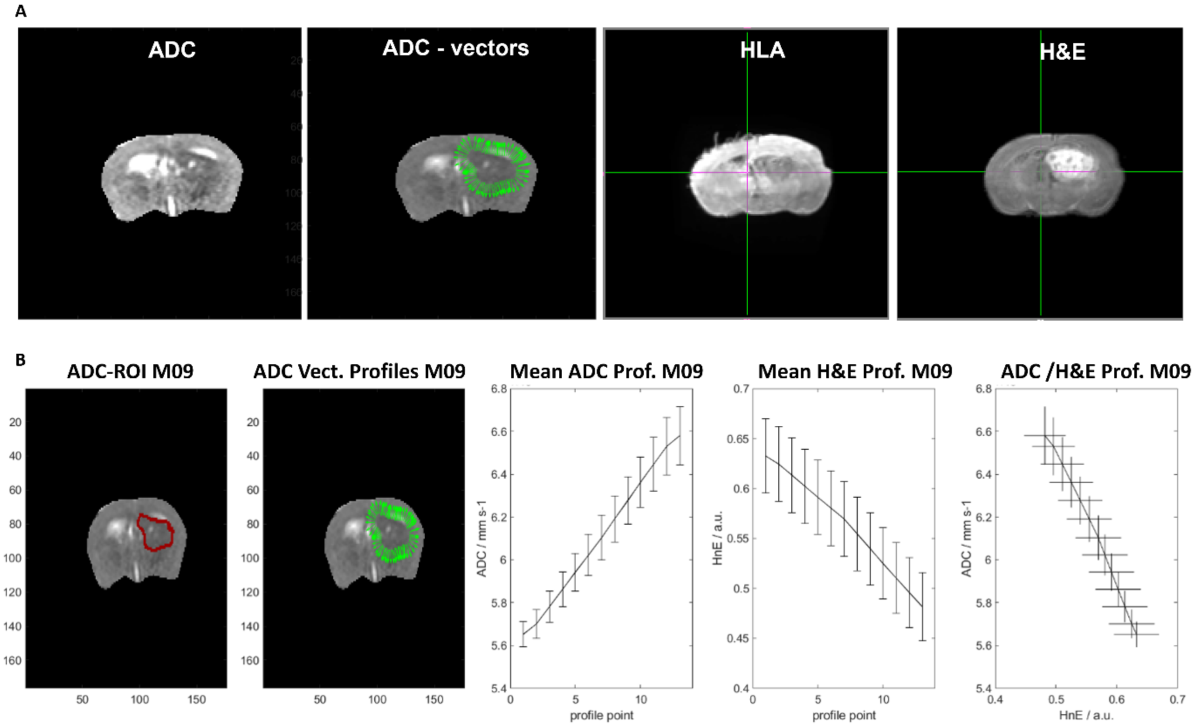

Fig. S8. T2W/ADC infiltration ratio assessment

As an additional proxy for the overall infiltrative extent of the tumour, we assessed the relationship between T2W/ADC ratio with the hypothesis that this ratio would increase with the extent of marginal tumour invasion. T2W/ADC was shown to increase linearly with the ADCvsTCD slope (Fig.S8A). Furthermore, T2W/ADC showed a negative relation to MDS reinforcing the hypothesis that ADC slope decreases with marginal invasion(Fig.S8B).

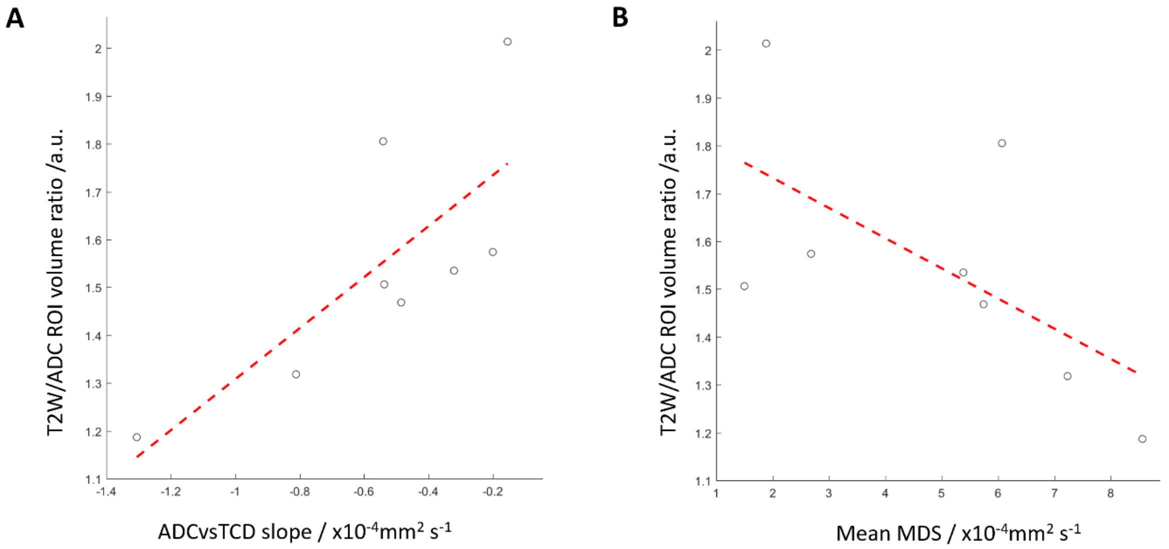

Fig. S9. Clinical study statistics summary

Survival Data

Statistic	Days	Months
Median OS	426	14.0
95% CI for median	382-721	12.5-23.7
Mean OS	516.5	17.0
Range	57-1696	1.9-55.7

Cox Regression

Model	HR	95% CI	p-value
MDS alone (per 1 SD)	0.47	0.25-0.89	**0.013**
MDS + Age: MDS	—	—	**0.010**
MDS + Age: Age	—	—	0.35
Schoenfeld PH test	—	—	0.76

Correlation

Statistic	Value
Pearson r (MDS vs OS)	0.636
95% CI	0.24-0.85
p-value	<0.005

Fig. S10. Necrotic pattern heterogeneity at the G7 tumour margins

Example of micro-necrosis pattens seen within the core of G7 model and becoming more sparse with distance from the injection point (yellow circle).

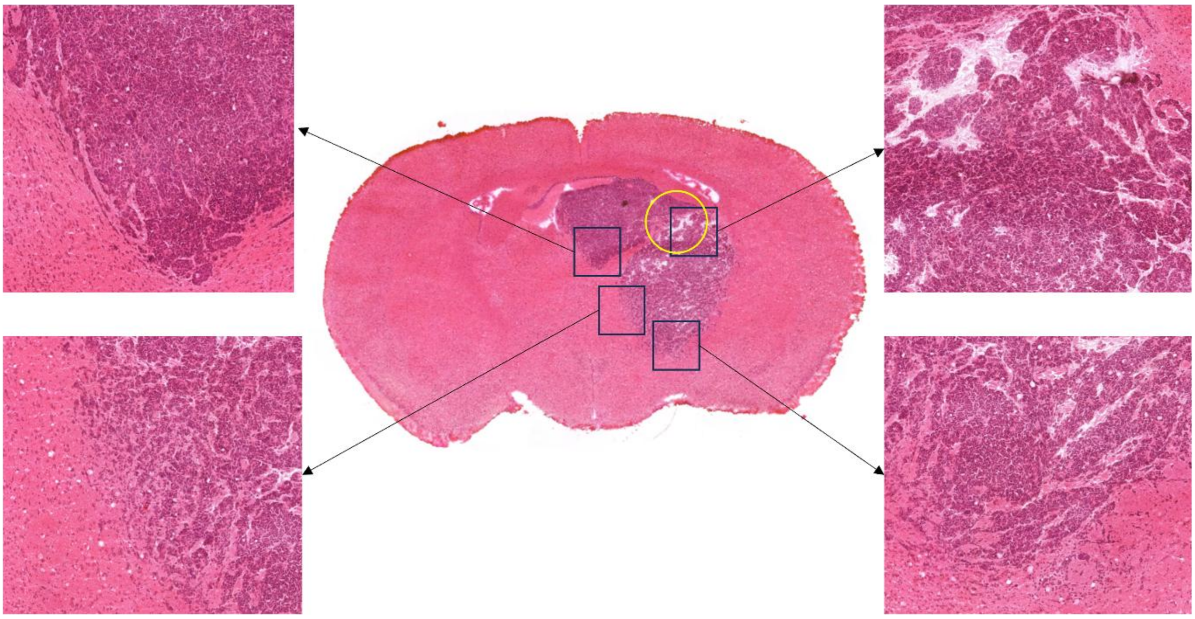

Supplement: vdag028_Supplementary_Data [file vdag028_supplementary_data.pdf]
